# Postoperative Pain following Root Canal Filling with Bioceramic vs. Traditional Filling Techniques: A Systematic Review and Meta-Analysis of Randomized Controlled Trials

**DOI:** 10.3390/jcm10194509

**Published:** 2021-09-29

**Authors:** Elina Mekhdieva, Massimo Del Fabbro, Mario Alovisi, Allegra Comba, Nicola Scotti, Margherita Tumedei, Massimo Carossa, Elio Berutti, Damiano Pasqualini

**Affiliations:** 1 Endodontics and Restorative Dentistry, CIR Dental School, Department of Surgical Sciences, University of Turin, 10126 Turin, Italy; elina.mekhdieva@unito.it (E.M.); mario.alovisi@unito.it (M.A.); allegra.comba@unito.it (A.C.); nicola.scotti@unito.it (N.S.); massimo.carossa@unito.it (M.C.); elio.berutti@unito.it (E.B.); 2 Department of Biomedical, Surgical and Dental Sciences, University of Milan, 20122 Milan, Italy; massimo.delfabbro@unimi.it (M.D.F.); margytumedei@yahoo.it (M.T.); 3 IRCCS Orthopedic Institute Galeazzi, 20161 Milan, Italy; 4 Department of Medical, Oral and Biotechnological Sciences, University “G. d′Annunzio” of Chieti-Pescara, 65122 Chieti, Italy

**Keywords:** meta-analysis, root canal filling, postoperative pain, bioceramic sealer, analgesic intake, flare-up

## Abstract

This meta-analysis aimed to evaluate postoperative pain (POP) following root canal filling (RCF) with gutta-percha/bioceramic sealer (BCS) vs. gutta-percha/traditional sealer (TS) techniques. Electronic databases were searched for randomized trials. Subgroup analyses were performed for analgesic intake, flare-ups, postoperative time (24/48 h), pulp status, and retreatment. The search yielded 682 records, and nine studies were selected. BCS was associated with significantly lower POP vs. TS at 24 h (*P* = 0.04) and 48 h (*P* = 0.0005). In addition, non-significant trends favoring BCS for analgesic intake at 24 h (*P* = 0.14), flare-ups (*P* = 0.24) and obturation techniques at 24 h (*P* = 0.41) and 48 h (*P* = 0.33), non-significant trends for lower POP with TS vs. BCS 24 h and 48 h in vital teeth (*P* = 0.50, *P* = 0.18, respectively), and for lower POP with BCS vs. TS in non-vital teeth at 24 h and 48 h (*P* = 0.16, *P* = 0.84, respectively). POP was numerically lower with TS vs. BCS at 24 h (*P* = 0.65) and 48 h after retreatment (*P* = 0.59). Moreover, POP did not vary between fillers when the treatment was over single (*P* = 0.28) or multiple visits (*P* = 0.50). BCS was associated with significantly lower short-term POP, and with a trend for lower analgesic intake and flare-up incidence, as compared to TS.

## 1. Introduction

Postoperative pain (POP) after root canal filling (RCF) affects up to 40% of patients [1]. The intensity and duration of POP vary according to multiple prognostic factors [2,3,4]. The filling technique is considered among the most relevant, in which warm vertical and cold lateral compaction as well as single cone are most traditionally utilized with resin-based or zinc-oxyde eugenol sealers [5,6]. The intensity and duration of postoperative pain are subjective and can be affected by many factors. In particular, by the severity of preoperative pain according to the medical history of the present diagnosis, tooth type, age, gender, etc. [2]. The intraoperative factors are also various, such as physical properties of the endodontic instrument used for the initial treatment, features of the irrigation protocol such as chemical solutions and concentrations, microbiological stability and resistance, histopathological state of the tissues surrounding the tooth, etc. [1,2,3,4]. At the final stage of root canal treatment during the obturation step, the endodontic sealer locally and directly contacts with the altered periapical tissues through the apical foramen and additional lateral canals. Accordingly, the physical and chemical properties of the sealer, such as pH-level, consistency, etc., also affect the intensity of postoperative endodontic pain [1,2]. The gutta-percha/bioceramic sealer (BCS) filling technique has gained popularity among endodontists due to features that include biocompatibility (due to their similarity with biological hydroxyapatite) and bioactive stimulation of periapical healing [7]. The setting time (30 min for working time), sealing ability, and antimicrobial properties are all key to the performance of endodontic sealers [8]. Premixed injectable formulations, preloaded syringes, and moldable putty forms are all available, facilitating ease of use [9]. However, there are no robust data evaluating any potential impact of BCS vs. traditional filling techniques on POP among randomized controlled trials (RCTs). The aim of this systematic review and meta-analysis was to assess the effect of the BCS filling technique compared with traditional filling techniques on POP in adult patients following RCF.

## 2. Materials and Methods

### 2.1. Study Design

This analysis considered all the studies that evaluated POP in adult patients, following RCF with BCS or traditional filling techniques.

Review question: How does the BCS filling technique affect the intensity of POP compared with the resin-based sealers (RBS) filling technique in patients undergoing a root canal treatment?

This study complies with the Preferred Reporting Items for Systematic Reviews and Meta-Analysis Statement (PRISMA), and was carried out on the basis of the Cochrane PICOS formula, defined as follows: *Population*, adult patients of both genders (not receiving analgesic or antibiotic medications, without long-term use of medications, not pregnant) with pulpal and/or periapical disease (without procedural errors, e.g., overfilling), who received an endodontic treatment in permanent teeth; *Intervention*, RCF with BCS; *Comparison*, RBS; *Outcome*, the primary outcome was the quality of life measured by the self-reported POP score; and *Study type*, RCTs. The systematic review protocol was registered with the International Prospective Register of Systematic Reviews (PROSPERO) a priori, ID: CRD42021227248.

### 2.2. Search Strategy and Inclusion Criteria

A comprehensive search strategy was designed to access biomedical databases (PubMed, Springer Link, DOSS, Scopus, Nature, Wiley Online Library, Web of Science Core Collection, BMJ, Cochrane Library, Oxford scholarship online, CINAHL complete, Access medicine, Science direct), grey literature (SIGLE—information on grey literature in Europe), and a clinical trials register (clinical trial.gov). A manual search of the main endodontic journals was also carried out (Journal of Endodontics, European Endodontic Journal, International Endodontic Journal). The search terms were “postoperative pain” AND “endodontic sealer” OR “root canal treatment” in studies published from January 2010 to January 2021 in English or German. The inclusion criteria were RCTs that assessed POP after RCF using the BCS filling technique in permanent teeth with pulpal and/or periapical disease. The selected studies compared the impact of BCS vs. TS on POP scores following RCF. POP scores could be reported using any self-recorded pain scale. We excluded the studies that did not compare the individual effect of endodontic sealer on the POP level; studies that additionally assessed the impact of anti-inflammatory medicines and laser applications; assessed the POP level after canal overfilling; or assessed the POP level after different root canal preparation techniques.

### 2.3. Study Selection

After the removal of duplicate records, the titles and abstracts of the identified studies were independently screened for eligibility by three reviewers (E.M., D.P., and M.D.F.). Consensus was achieved through discussion, where there was discordance in study selection. 

### 2.4. Data Extraction

Three reviewers (E.M., D.P., and M.D.F.) independently extracted data from studies that met the inclusion criteria, using a standardized data collection table consisting of strings: References (title, authors, year of publication, country), study design, sample size, age/sex groups, inclusion and exclusion criteria, diagnosis, pre-op status, operator, quantity of visits, glidepath, instrumentation, irrigation protocols, obturation technique and materials, restoration, POP assessment time and scale, analgesics intake, flare-up, etc. If multiple treatment groups were presented, the data conforming to PICO were collected. Moreover, if any information was missed, the authors were contacted through personal communication via e-mail. Furthermore, if there was no response for up to 5 weeks, the study was not included in the meta-analysis.

### 2.5. Quality Assessment

The quality of each RCT was assessed according to the Cochrane Risk of Bias Tool. All the domains (random sequence generation, allocation concealment, performance bias, blinding of outcome, attrition bias, reporting bias) were rated as “high”, “low” or “unclear” risk of bias. We set an additional risk of bias according to the “Operator” (expert endodontist: Low risk; undergraduate student: High risk). Studies were classified as overall high risk if they contained one or more domains rated as high risk; overall moderate risk if they contained no high-risk domain and one or more were judged as unclear; and overall low risk if all the domains were judged at a low risk of bias.

### 2.6. Meta-Analysis

The general methodology of this review followed the directions of the Cochrane Handbook for Systematic Reviews of Interventions [10]. If possible, the odds ratio (OR)/risk ratio (RR) or standardized mean differences (SMD) and their 95% confidence intervals (CI) were calculated for the quantitative data extracted from each RCT. Results from comparable groups of studies were pooled into a meta-analysis using Review Manager (RevMan) Software (version 5.4.1, The Cochrane Collaboration, 2020). The findings are presented in a narrative form, if statistical pooling was not possible. The subgroup analysis was conducted on parameters reported by at least two studies. The significance of any variation and degree of heterogeneity was determined by *I²* and chi-square statistics, respectively. Publication bias tests were not conducted due to the low number of studies included.

In some cases, the included studies will present with peculiar features (for example, different inclusion criteria with respect to other studies, presence of patients with systemic conditions or asymptomatic patients). Moreover, the sensitivity analysis will be performed to assess if the exclusion of the study will affect the outcome of the analysis.

## 3. Results

### 3.1. Study Selection

**Figure 1:**
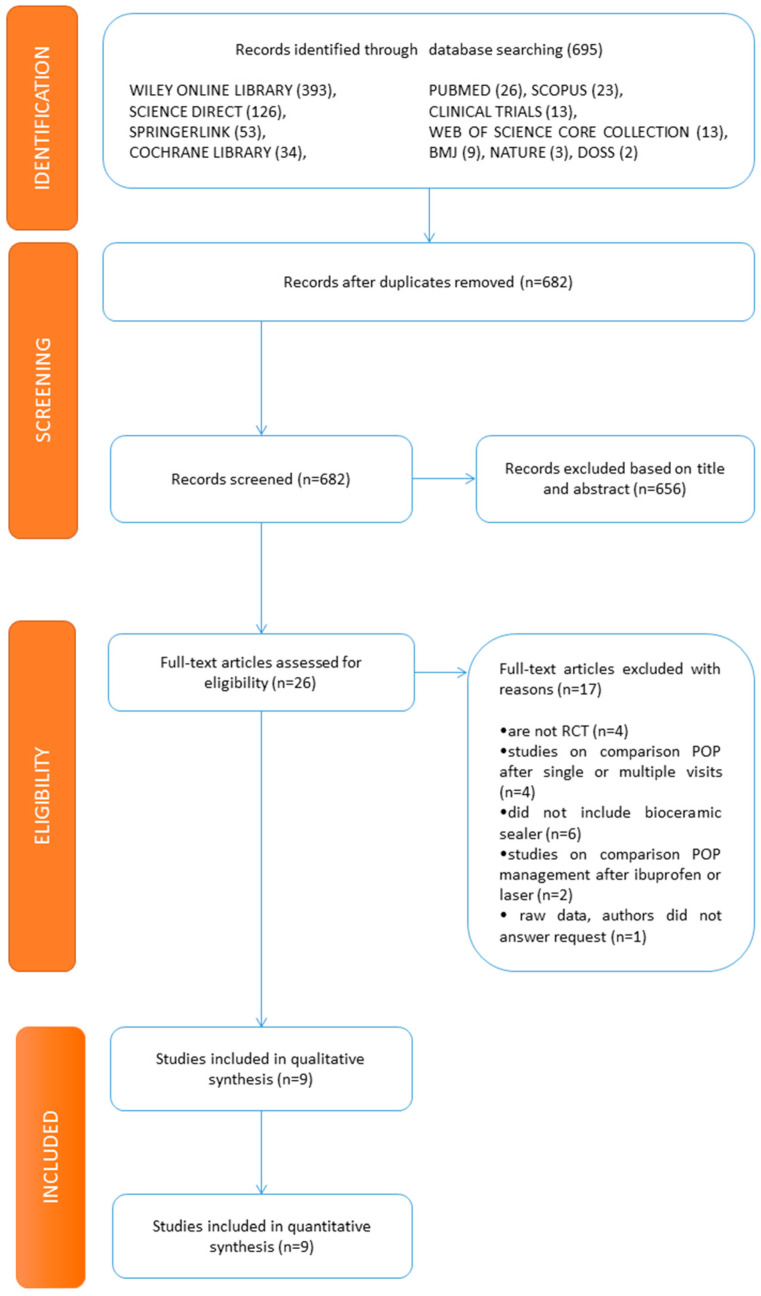
The preferred reporting items for systematic reviews and meta-analysis flow diagram of the search results.

The search strategy identified 695 records, including 13 duplicates. The remaining 682 records were screened by the title and abstract (Figure 1). In total, 656 records were considered irrelevant and removed, leaving 26 studies that were assessed for eligibility by full-text reviewing. At this stage, 17 studies were excluded [11,12,13,14,15,16,17,18,19,20,21,22,23,24,25,26,27], most commonly since they did not include a BCS (Supplementary Materials Table S1). Finally, nine studies were included for systematic review. Although each of the selected studies evaluated the POP level following different filling techniques, the variability within the study designs and the materials and methods employed required specific consideration during the analysis.

### 3.2. Study Characteristics

The characteristics of the selected RCTs are presented in Table 1. All the studies were published single-center RCTs that reported the characteristics of teeth, pre-operative status, diagnosis, instrumentation details and irrigation protocols, endodontic sealers and filling techniques used, analgesic intake, incidence of flare-ups, number of visits, and pulp and periapical status.

All the selected studies included teeth with pulp or periapical pathologies, without signs of radiolucency, requiring a primary endodontic treatment. However, only three studies included teeth that needed retreatment [28,29,30]. Four studies included teeth that were asymptomatic pre-operatively [30,31,32,33], two studies only included symptomatic teeth [28,34], and three studies examined asymptomatic and symptomatic teeth [29,35,36]. Only one study managed an endodontic treatment without local anesthesia [31]. Furthermore, three studies assessed POP in anterior single-rooted teeth only [31,32,33].

The instrumentation and irrigation protocols were similar across the studies, but the filling techniques varied: Warm vertical condensation (WVC) was utilized in five studies, single-cone technique (SCT) in three studies, carrier-based obturation in one study, and lateral condensation in one study. The resin-based sealer (RBS) was utilized as a control group in all the included studies. One study deliberately carried out the filling procedure during a second visit to exclude the influence of instrumentation stage on POP. The other eight studies evaluated POP in the context of a single visit treatment.

A variety of pain rating scales were used, including variations of the Visual Analog Scale and Verbal Rating Scale, as well as the Heft and Parker Pain Rating Scales of 0–10, 0–100, 0–170 or verbal (no pain/mild pain/moderate pain/severe pain). The data were reported as either means or percentages. Four studies reported an analgesic intake and three studies reported an incidence of flare-ups.

Remarkably, none of the included studies identified significant differences in the POP level, analgesic intake or incidence of flare-ups between different endodontic sealers.

### 3.3. Risk of Bias

The risk of bias in the nine RCTs is summarized in Table 2. One study was scored as having an overall “low” risk of bias, six studies as having an overall “moderate” risk, and two studies as having an overall “high” risk of bias, since the treatment was managed by undergraduate students.

### 3.4. Meta-Analysis

**Figure 2:**
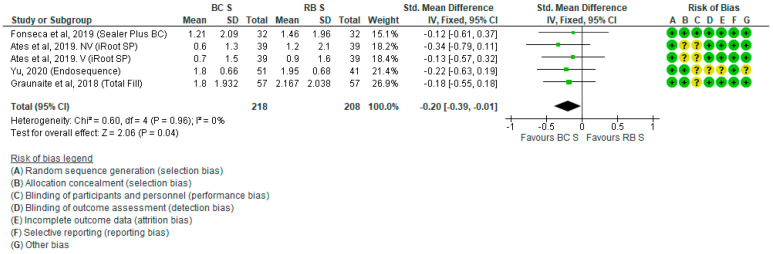
Forest plot of POP level 24 h after RCF with BCS vs. RBS.

**Figure 3:**
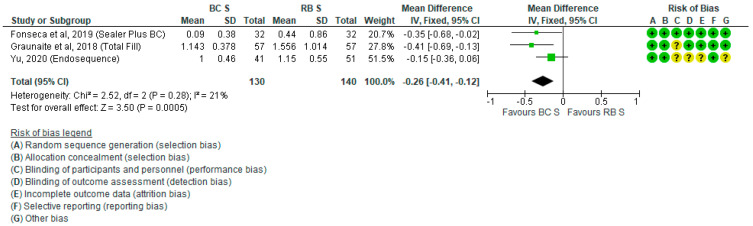
Forest plot of POP level 48 h after RCF with BCS vs. RBS.

Pooled POP data (mean ± standard deviation [SD]) experienced by patients 24 h and 48 h after RCF with BCS or RBS are presented in Figure 2 and Figure 3, respectively. Six studies did not report the mean POP ± SD 48 h after RCF and these were not included in the respective forest plot [28,30,33,34,35,36]. Since in the study by Graunaite et al. 2018 [31] asymptomatic patients were treated, as opposed to all the other studies in which patients were symptomatic, the sensitivity analysis was performed in all analyses where that study was considered, to see if the results changed with the exclusion of asymptomatic subjects. Pooled data analyses indicate that POP was significantly lower in patients who underwent RCF with BCS compared with RBS at 24 h (SMD = −0.20; *P* = 0.04) and 48 h (SMD = −0.26; *P* = 0.0005) after treatment. After the sensitivity analysis, by excluding Graunaite et al. 2018, the results did not change significantly.

**Figure 4:**
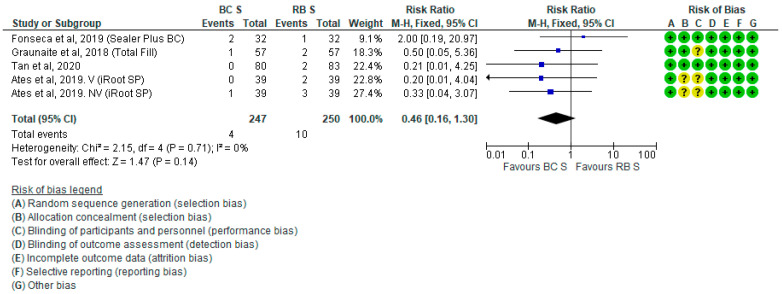
Forest plot of analgesic intake 24 h after RCF with BCS vs. RBS.

The analgesic intake did not significantly differ between the BCS and RBS groups 24 h after RCF (RR = 0.46; *P* = 0.14; Figure 4). Five studies were not included in this forest plot due to a lack of available data [11,12,13,33,36]. The incidence of flare-up was also not significantly different between the BCS and RBS groups (OR = 0.32; *P* = 0.24; Supplementary Materials Figure S1). After the sensitivity analysis, by excluding Graunaite et al. 2018, the results did not change significantly.

The next two diagrams underline the pain prevalence and severity of the BCS group over the RBS group in 24 h (Supplementary Materials Figure S2) and 48 h (Supplementary Materials Figure S3) after RCF.

The probability of “No pain” 24 h after treatment was 1.12× higher in the BCS group vs. the RBS group (OR = 1.12; 95% CI, 0.77–1.64; *P* = 0.86), while the same was observed for “Moderate pain” probability (OR = 1.21; 95% CI, 0.61–2.38; *P* = 0.59). There was no heterogeneity in the study effect for the BCS and RBS groups (*I²* = 0%; *P* = 0.86, and *I²* = 0%; *P* = 0.89, respectively), indicating perfect consistency in the results. The probability of “Mild pain” and “Severe pain” was 1.2× and 1.7× higher in the RBS group vs. the BCS group, respectively (OR = 0.83; 95% CI, 0.54–1.25; *P* = 0.37, and OR = 0.59; 95% CI, 0.08–4.58; *P* = 0.62). There was also no heterogeneity in the study effect in either group (*I²* = 0%; *P* = 0.97, and *I²* = 0%; *P* = 0.61, respectively; Supplementary Materials Figure S2).

A diagram of pain characteristics in the BCS and RBS groups 48 h after treatment is presented in Supplementary Materials Figure S3. The studies that did not report pain characteristics 48 h after treatment were excluded. The probability of “No pain” 48 h after treatment was 1.21× higher in the BCS group vs. the RBS group (OR = 1.21; 95% CI, 0.60–2.42; *P* = 0.60; heterogeneity: *I²* = 18%; *P* = 0.30). “Mild pain” was more commonly reported in the RBS group vs. the BCS group 48 h after treatment (OR = 0.82; 95% CI, 0.40–1.68; *P* = 0.59; heterogeneity: *I²* = 1%; *P* = 0.40), while “Moderate pain” was equally likely in both groups 48 h after treatment (OR = 1.00; 95% CI, 0.14–7.27; *P* = 1.00; heterogeneity: *I²* = 0%; *P* = 0.33). “Severe pain” was reported only once in both groups (Supplementary Materials Figure S3). After the sensitivity analysis, by excluding Graunaite et al. 2018, the results did not change significantly neither for data after 24 h nor for 48 h after RCT.

The effect of the obturation technique (WVT vs. SCT) on POP based on data 24 h and 48 h after RCF is presented in Supplementary Materials Figures S4 and S5. The studies that did not report 48-h data were excluded. There was a numerical difference in POP in favor of BCS for both WVT and SCT subgroups 24 h after RCF (OR = 0.85; *P* = 0.41). The probability of POP was numerically higher in the RBS subgroup that underwent the WVT technique (*P* = 0.49) in 1.2× and lower in the BCS subgroup that underwent the SCT technique (*P* = 0.64; Supplementary Materials Figure S4). There was evidence of lower POP in both WVT and SCT subgroups of the BCS group 48 h after RCF (*P* = 0.33). The probability of POP did not differ according to the obturation technique in the BCS group, but was 1.3× higher in the RBS group 48 h after RCF (OR = 0.78; *P* = 0.33; Supplementary Materials Figure S5).

The probability of POP by the pulp status (vital [V] and non-vital [NV] pulp) 24 h and 48 h after RCF is presented in Supplementary Materials Figures S6 and S7, respectively. The studies that did not report 48-h data were excluded. There was evidence of a non-significant difference in POP in favor of RBS in the V subgroup (*P* = 0.50) and a trend for lower POP in NV teeth within the BCS group 24 h after RCF (*P* = 0.16). However, there was no statistically significant overall effect of V vs. NV pulp on POP (OR = 0.84; *P* = 0.45; Supplementary Materials Figure S6). The probability of POP was 2× higher in the V pulp of the BCS group, and there was evidence of lower POP in the V subgroup of the RBS group 48 h after RCF (OR = 2.01; *P* = 0.18). There was also a non-significant trend for lower POP in NV teeth within the BCS group (OR = 0.92; *P* = 0.84; Supplementary Materials Figure S7). After the sensitivity analysis, by excluding Graunaite et al. 2018, the results did not change significantly.

Pooled data from the three studies that reported the retreatment show a trend for a difference in POP in favor of RBS (OR = 1.20; *P* = 0.65) 24 h after RCF (Supplementary Materials Figure S8). The meta-analysis determined that there was a 48% level of heterogeneity within the included nine studies. Only one study had analyzed a consistent number of cases [31]. Supplementary Materials Figure S9 presents POP in retreatment groups 48 h after RCF. Unfortunately, the limited numbers of studies and retreated cases are insufficient to determine any effect of BCS vs. RBS on POP (OR = 1.34).

There was no significant difference in POP between the BCS and RBS groups when the treatment was carried out over single (OR = 0.77; *P* = 0.28) or multiple visits 24 h after RCF (OR = 0.81; *P* = 0.50; Supplementary Materials Figure S10). A lack of data precluded the equivalent analysis of POP 48 h after RCF.

## 4. Discussion

This meta-analysis of nine pooled RCTs indicates that POP was significantly lower after RCF with BCS compared with RBS. However, none of the RCTs individually reported any significant effect of BSC vs. RBS on POP [28,29,30,31,32,33,34,35,36].

In this analysis, we found that BCS was non-significantly correlated with reduced analgesic intake vs. RBS, an observation that was also reported by one of the included studies [35] using the warm-obturator filling technique. However, two of the other included RCTs [32,33] demonstrated comparable analgesic intake in the control and experimental groups after RCF using SCT.

This systematic review found a non-significant trend of reduced flare-up in the BCS group vs. the RBS group. This is supported by one of the included RCTs that reported a significant reduction of flare-up following RCF with BCS vs. RBS [35]. However, another reported an equal occurrence between groups [31].

Regarding the warm and cold filling techniques, SCT with BCS has previously been associated with higher POP, while WVT with RBS has been associated with the lowest POP scores [28]. However, our pooled analysis suggests that there is a non-significant trend in favor of BCS.

Our results indicate that POP was lower in the V pulp when filled with RBS and in the NV pulp when filled with BCS. However, we found no additional background literature to place this in context.

Our results also evidence a non-significant difference in POP in favor of RBS at retreatment. Moreover, the only RCT [31] included to report this parameter indicates no difference between filling techniques in POP, following the retreatment procedures.

According to our results, the trend for lower POP following RCF with BCS vs. RBS filling technique was observed across single and multiple visit treatments. However, there are no studies in the literature to provide additional context.

The main limitations of this review are inter-study variability and inconsistency, as well as a lack of clinically relevant outcomes. Furthermore, as mentioned, different scales for pain measurement were used in different studies. Though the authors made efforts to resize all the scales to a 1–10 scale, it is difficult to understand if this had a relevance in the results. Of course, for future studies, it is recommended to use only scales for which there is an overall consensus. Therefore, the findings presented here need to be confirmed by further well-designed studies and should be interpreted with caution.

## 5. Conclusions

Our findings suggest that the BCS filling technique may positively affect POP, while there was a trend of a beneficial effect for analgesic intake, incidence of flare-up, pulp status, and number of visits when using BCS, compared with RBS. However, due to several limitations in these analyses, further well-designed clinical studies are warranted to supplement our results.

## Figures and Tables

**Table 1:** Cochrane PICO formula.

Patients	Intervention	Comparison	Outcome
With pulp / periapical disease	Filling with bioceramic technique	Filling with traditional technique	Postoperative pain

**Table 2:** Summary of the risk of bias of the included studies.

Study	Risk of Bias							
	A	B	C	D	E	F	G	Overall
GRAUNAITE et al. 2018 [31]	+	+	?	+	+	+	+	?
PAZ et al. 2018 [28]	?	?	?	+	+	+	-	-
ATES et al. 2019 [35]	+	?		+	+	+	+	?
FERREIRA et al. 2019 [33]	+	+	+	+	+	+	?	?
FONSECA et al. 2019 [32]	+	+	+			+	+	+
NABI et al. 2019 [34]	?	?	+	+	+	+	?	?
SHARMA et al. 2019 [36]	?	?	?	?	+	+	?	?
TAN et al. 2020 [30]	+	+	+	+	+	+	+	+
YU 2020 [29]	+	+	?	?	?	+	?	?

“+”: low risk of bias, “?”: unclear risk of bias,“-”: high risk of bias. (**A**) Random sequence generation (selection bias). (**B**) Allocation concealment (selection bias). (**C**) Blinding of participants and personnel (performance bias). (**D**) Blinding of outcome assessment (detection bias). (**E**) Incomplete outcome data (attrition bias). (**F**) Selective reporting (reporting bias). (**G**) Other bias.

## Supplementary Materials

The following are available online at https://www.mdpi.com/article/10.3390/jcm10194509/s1, Table S1: Excluded studies with reasons for exclusion, Figure S1: Forest plot of flare-up after RCF with BCS vs. RBS, Figure S2: Diagram of pain characteristics summary in a subgroup of BCS and RBS traditional filling technique groups 24 h after RCF, Figure S3: Diagram of pain characteristics summary in a subgroup of BCS and RBS traditional filling technique groups 48 h after RCF, Figure S4: Forest plot of POP in patients treated with warm and cold filling techniques 24 h after RCF with BCS vs. RBS, Figure S5: Forest plot of POP in patients treated with warm and cold techniques 48 h after RCF with BCS vs. RBS, Figure S6: Forest plot of POP in vital and non-vital pulp 24 h after RCF with BCS vs. RBS, Figure S7: Forest plot of POP in vital and non-vital pulp 48 h after RCF with BCS vs. RBS, Figure S8: Forest plot of POP following retreatment 24 h after RCF with BCS vs. RBS, Figure S9: Forest plot of POP following retreatment 48 h after RCF with BCS vs. RBS, Figure S10: Forest plot of POP 24 h after RCF with BCS vs. RBS following a single or multiple visit treatment.
