# Supplementary material for: Postoperative Pain following Root Canal Filling with Bioceramic vs. Traditional Filling Techniques: A Systematic Review and Meta-Analysis of Randomized Controlled Trials
Source: J Clin Med. 2021 Sep 29;10(19):4509. doi: 10.3390/jcm10194509 (PMC8509229; doi:10.3390/jcm10194509)

Table S1. Excluded studies with reasons for exclusion.

№	*Study*	*Reason for Exclusion*
1	Technical quality and postoperative pain of single visit endodontic treatment of chronic apical periodontitis filled by bioceramic sealer<br>*S.O. Niang, K. Bane, M. Sarr, B. Tourè, and P. Machtou*<br>*IP Indian J Conserv Endod 2018;3:92–7*	Not a RCT
2	Postoperative pain in cold and warm endo-techniques using bioceramic sealer<br>*I. Filipov, P. Zagorchev, S. Dimitrova, and N. Manchorova-Veleva*<br>*International Association for Dental Research 2018 Poster session*	Raw data, authors did not answer request
3	Postoperative pain and associated factors in patients undergoing single visit root canal treatment on teeth with vital pulp<br>*R. Shresha, D. Shresha, and R. Kayasatha*<br>*Kathmandu Univ Med 2018;16:220–3.*	Not a RCT
4	Predictive models of pain following root canal treatment: a prospective clinical study<br>*A. Arias, J.C.de la Macorra, J.J. Hidago, and M.Azabal*<br>*Int Endod J 2013;46:784–93*	Not a RCT
5	Comparison of post-obturation pain experience following one-visit and two-visit root canal treatment on teeth with vital pulps: a randomized controlled trial<br>*C. Wang, P. Xu, L. Ren, G. Dong, and L.Ye*<br>*Int Endo J 2010; 43:692–7*	Study comparing POP after single or multiple visits

6	Factors associated with postoperative pain in endodontic therapy<br>*D. Sadaf, M.Z. Ahmad*<br>*Int J BioMed Sci 2014;10:243–7*	Not a RCT
7	Comparative evaluation of pain , tenderness and swelling followed by radiographic evaluation of periapical changes at various intervals of time following single and multiple visit endodontic therapy: an in vivo study<br>*M.B. Prashanth, P.N. Tavane, S. Abraham, and L. Chacko*<br>*J Contemp Dent Pract 2011;12:187–91*	Did not include BCS
8	Assessment of pain and dissolution of apically extruded sealers and their effect on the periradicular tissues<br>*G. Shashirekha, A. Jena, S. Pattanaik, and J. Rath*<br>*J Conserv Dent 2018;21:546–50*	Did not include BCS
9	Comparison of the effect of photobiomodulation therapy and Ibuprofen on postoperative pain after endodontic reatment: randomized, controlled clinical study<br>*E. Costa Nunes, F.J. Herkrath, E. Hideki Suzuki, E. C. Gualberto Junior, A.A.F. Marques, and E.C. Sponchiado Junior*<br>*Lasers Med Sci 2020;35:971–8*	Study comparing POP management after ibuprofen or laser
10	The effect of pulpotomy using a Calcium-enriched mixture cement versus one-visit root canal therapy on postoperative pain relief in irreversible pulpitis: a randomized clinical trial<br>*S. Asgary, and M.J. Eghbal*	Did not include BCS

	Odontology 2010;98:126–33	
11	Post-obturation pain following one-visit and two-visits root canal treatment in necrotic anterior teeth<br>*K.N. Rao, R. Kandaswamy, G. Umashetty, V.P. Singh Rathore, C. Hotkar, and B.S. Patil*<br>*J Int Oral Health 2014;6:28–32*	Study comparing POP after single or multiple visits
12	Influence of photodynamic therapy in the control of postoperative pain in endodontic treatment: a cross-sectional randomized clinical trial<br>*F.M.S. Yoshinari, K.F. Souza Pereira, D. Zafalon Beraldo, J.C.L. da Silva, E.J. Zafalon, and P.G. da Silva*<br>*Pesqui Bras Odontopediatria Clin Integr 2019;191:43*	Study comparing POP management after ibuprofen or laser
13	Postoperative pain intensity after single- versus two-visit nonsurgical endodontic retreatment: a randomized clinical trial<br>*Y.E. Hepsenoglu, T.F. Eyuboglu, and M. Ozcan*<br>*J Endod 2018;44:1339–46*	Study comparing POP after single or multiple visits
14	A prospective randomized comparative study of cold lateral condensattion versus core/gutta-percha in teeth with periapical lesions<br>*G.K. Demirci, and M.K. Chalishkan*<br>*J Endod 2016; 42:206–10*	Did not include BCS
15	Maintaining apical patency does not increase postoperative pain in molars with necrotic pulp and apical periodontitis: a randomized controlled trial<br>*I.E. Yaylali, S. Kurnaz, and Y. Tunca*<br>*J Endod 2018;44:335–40*	Did not include BCS

16	A randomized controlled trial to evaluate the incidence of postoperative pain and flare-ups in single and multiple visits root canal treatment<br>*V.K. Dhyani, S. Chhabra, V.K. Sharma, and A. Dhyani*<br>*Med J Armed Forces India 2020*	Study comparing POP after single or multiple visits
17	Comparative evaluation of postoperative pain after root canal treatment using three different sealers, Viz., Tubli-Seal EWT, Apexit Plus, AH Plus: an in-vivo study<br>*B. Gudlavalleti, and A.A. Patil*<br>*J Clin Diagn Res 2020;14:ZC04–9*	Did not include BCS

POP, postoperative pain; RCT, randomized clinical trial.

Figure S1. Forest plot of flare-up after RCF with BCS vs. RBS.

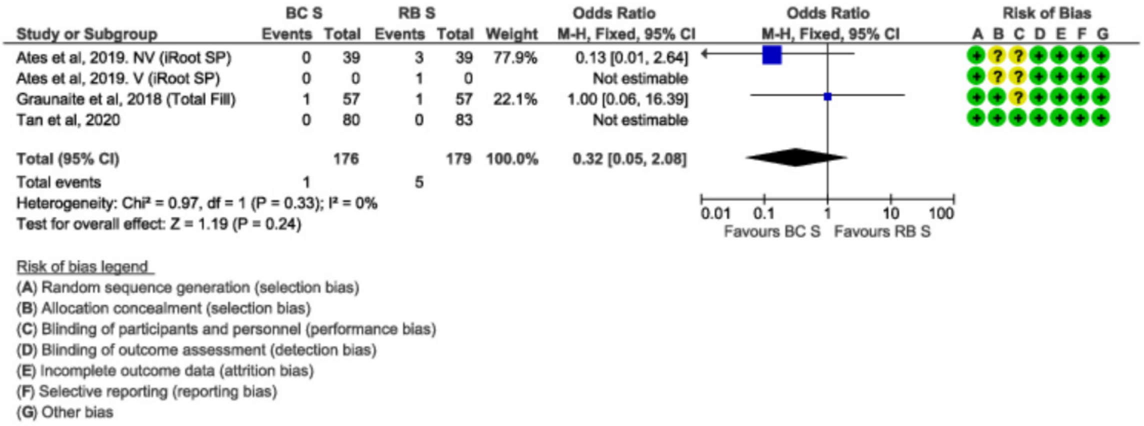

Figure S2. Diagram of summary of pain characteristics in a subgroup of BCS and RBS traditional filling technique groups 24 h after RCF.

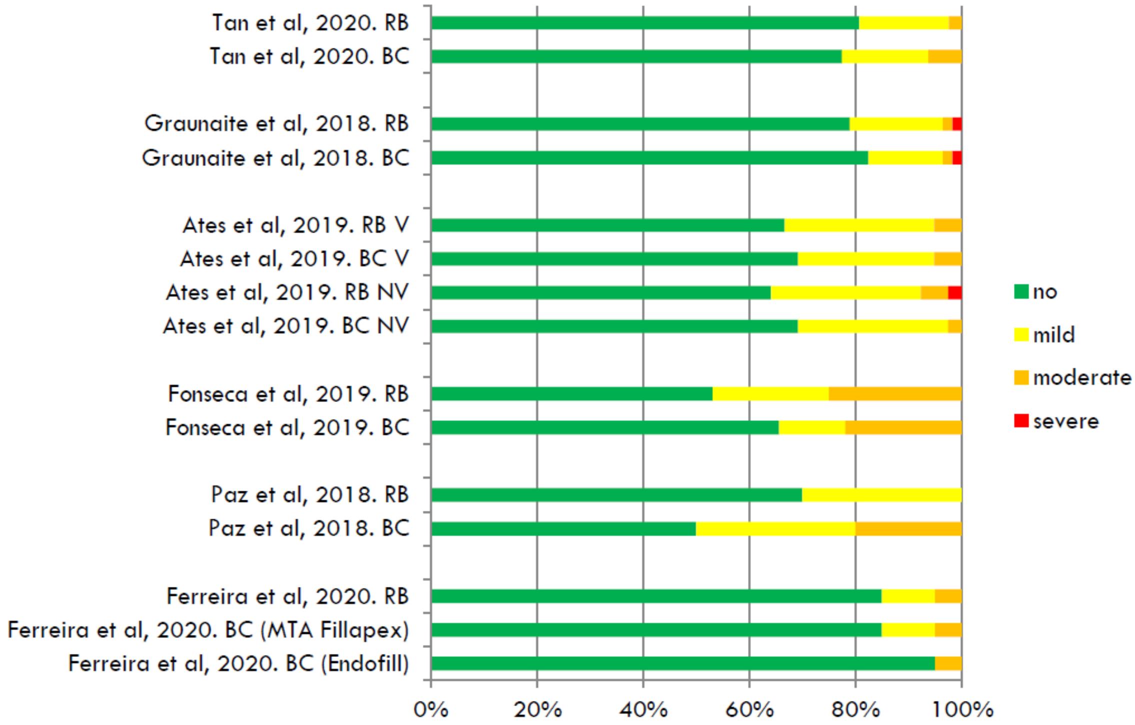

Figure S3. Diagram of summary of pain characteristics in a subgroup of BCS and RBS traditional filling technique groups 48 h after RCF.

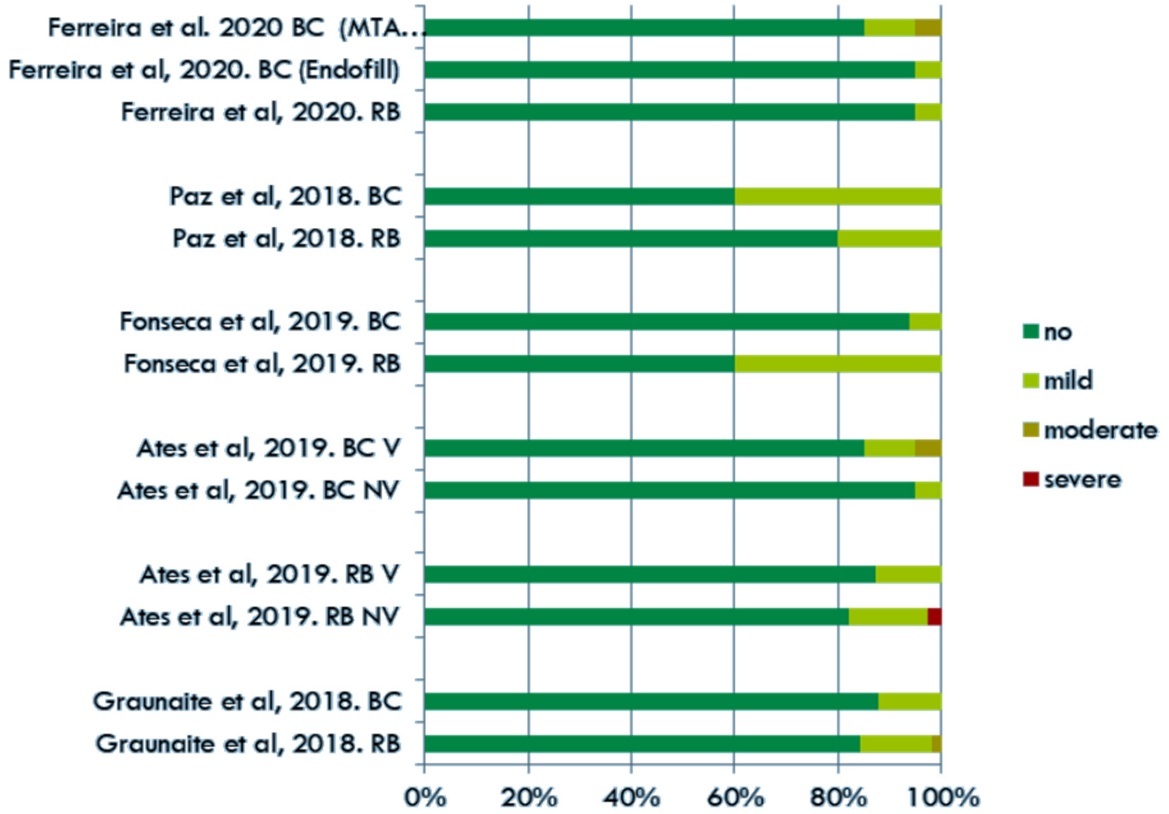

Figure S4. Forest plot of POP in patients treated with warm and cold filling techniques 24 h after RCF with BCS vs. RBS.

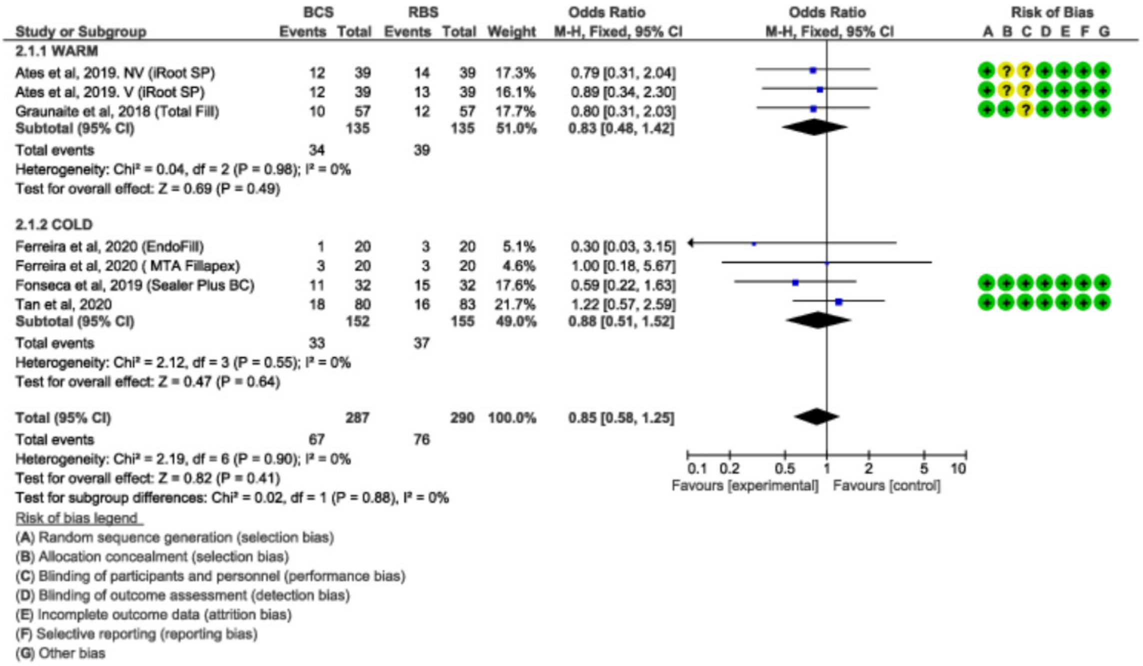

Figure S5. Forest plot of POP in patients treated with warm and cold techniques 48 h after RCF with BCS vs. RBS.

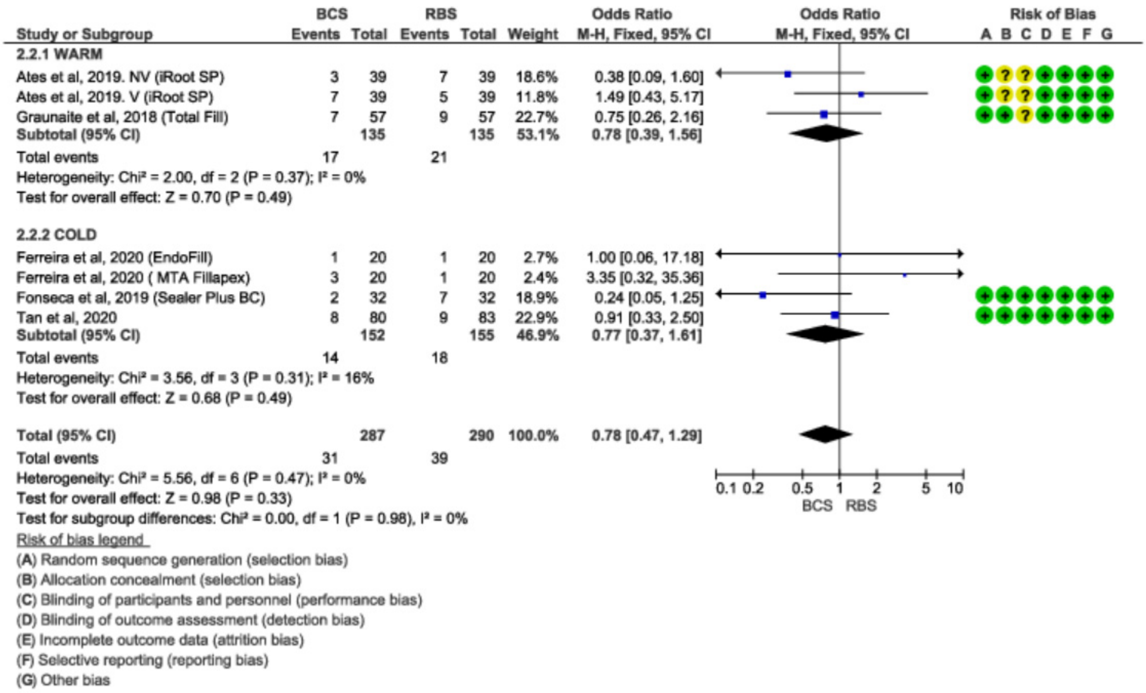

Figure S6. Forest plot of POP in vital and non-vital pulp 24 h after RCF with BCS vs. RBS.

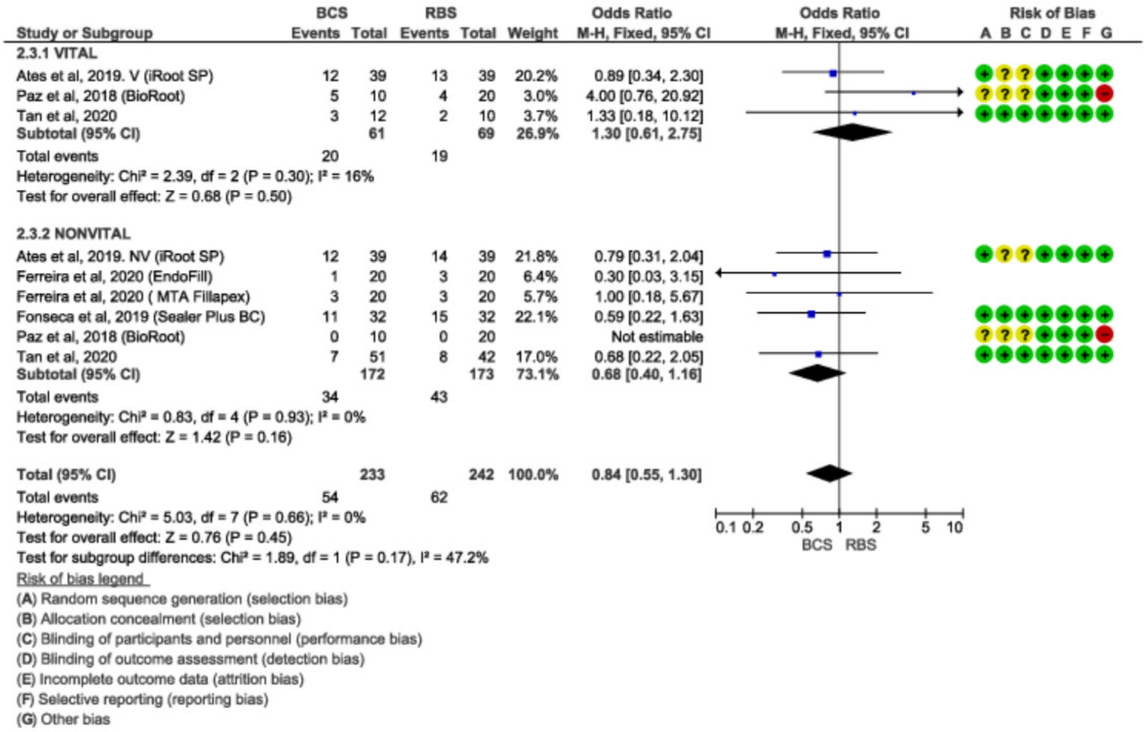

Figure S7. Forest plot of POP in vital and non-vital pulp 48 h after RCF with BCS vs. RBS.

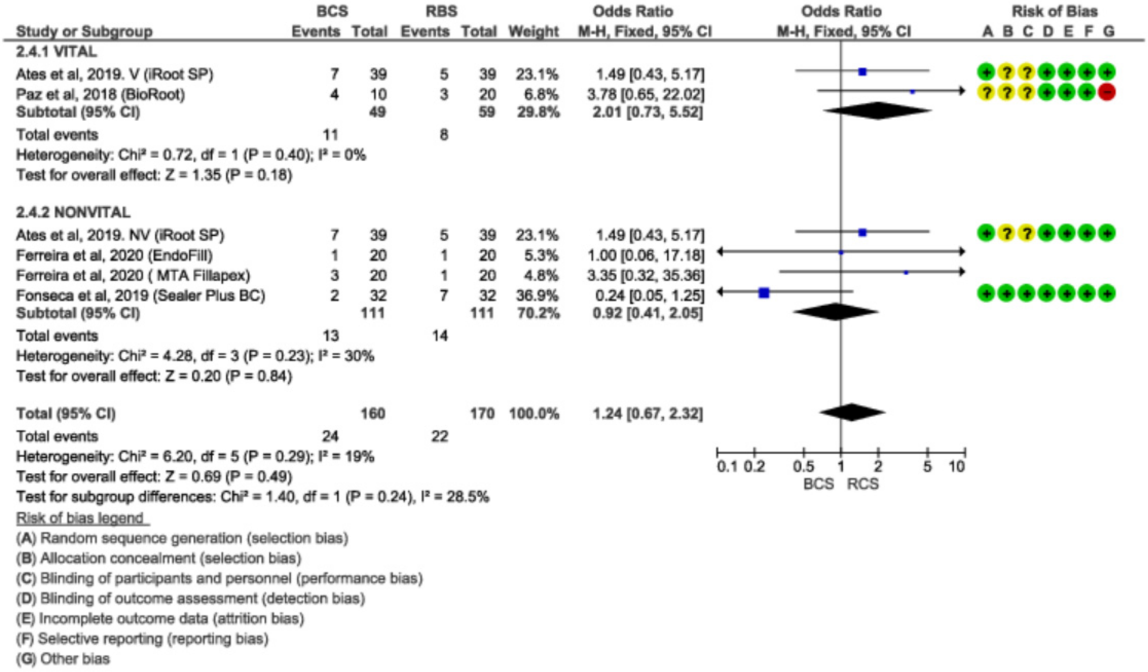

Figure S8. Forest plot of POP following retreatment 24 h after RCF with BCS vs. RBS.

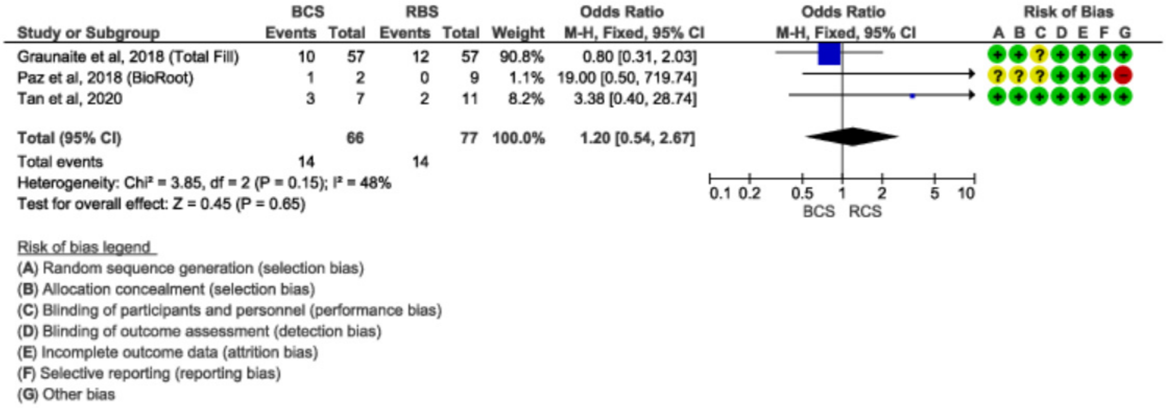

Figure S9. Forest plot of POP following retreatment 48 h after RCF with BCS vs. RBS.

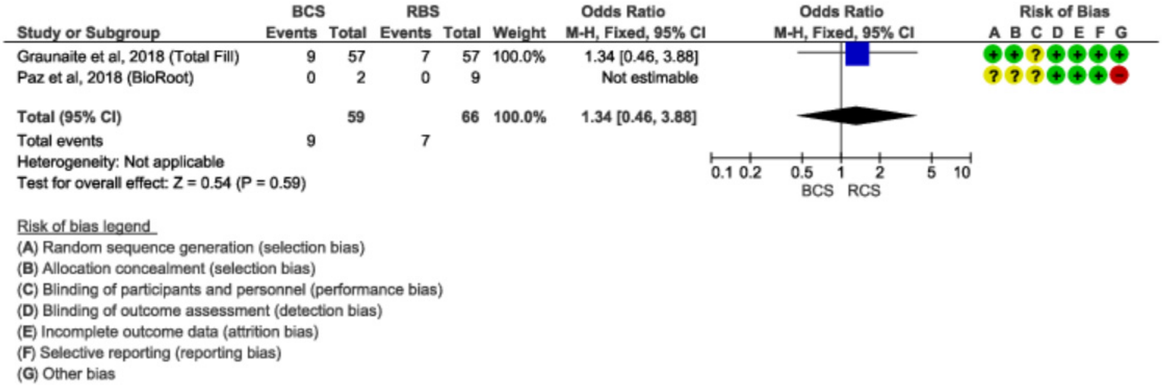

Figure S10. Forest plot of POP 24 h after RCF with BCS vs. RBS following single or multiple visit treatment.

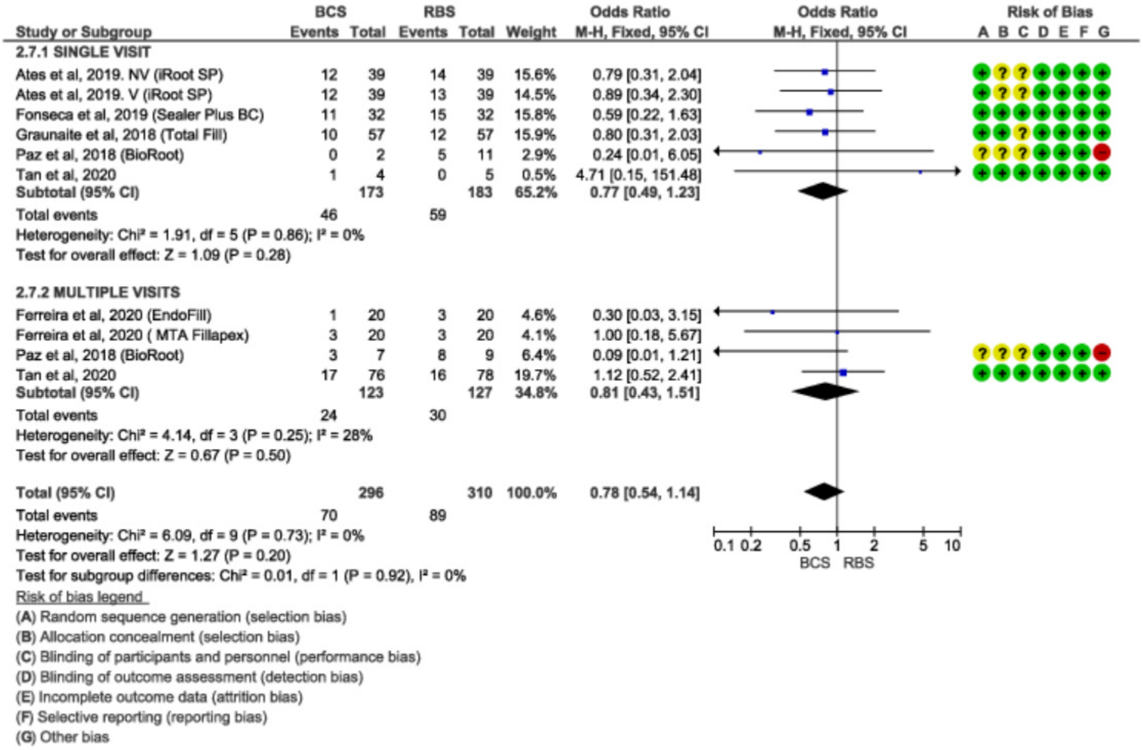

Supplement: Supplementary file 1 [file jcm-10-04509-s001.zip › Supplementary updated.pdf]
